# The Role of Regulator-Imposed Post-Approval Studies in Health Technology Assessments for Conditionally Approved Drugs

**DOI:** 10.34172/ijhpm.2020.198

**Published:** 2020-10-27

**Authors:** Rick A. Vreman, Lourens T. Bloem, Stijn van Oirschot, Jarno Hoekman, Menno E. van der Elst, Hubert GM Leufkens, Olaf H. Klungel, Wim G. Goettsch, Aukje K. Mantel-Teeuwisse

**Affiliations:** ^1^Division of Pharmacoepidemiology and Clinical Pharmacology, Utrecht Institute for Pharmaceutical Sciences, Utrecht University, Utrecht, The Netherlands.; ^2^The National Health Care Institute, Diemen, The Netherlands.; ^3^Dutch Medicines Evaluation Board, Utrecht, The Netherlands.; ^4^Department of Innovation Studies, Copernicus Institute of Sustainable Development, Utrecht University, Utrecht, The Netherlands.; ^5^Faculty of Health Sciences, University of Southern Denmark, Odense, Denmark.

**Keywords:** Conditional, Authorization, Health Technology Assessment, Post-approval, Relative Effectiveness, Evidence

## Abstract

**Background: **The European Medicines Agency (EMA) aims to resolve uncertainties associated with conditionally approved drugs by imposing post-approval studies. Results from these studies may be relevant for health technology assessment (HTA) organizations. This study investigated the role of regulator-imposed post-approval studies within HTA.

**Methods:** For all conditionally approved drugs up to December 2018, regulator-imposed post-approval studies were identified from EMA’s public assessment reports. The availability for and inclusion of study results in relative effectiveness (re)assessments were analyzed for 4 European HTA organizations: NICE (National Institute for Health and Care Excellence, England/Wales), HAS (Haute Autorité de Santé, France), ZIN (Zorginstituut Nederland, the Netherlands) and the European Network for Health Technology Assessment (EUnetHTA, Europe). When study results became available between an HTA organization’s initial assessment and reassessment, it was evaluated whether and how they affected the assessment and its outcome.

**Results: **For 36 conditionally approved drugs, 98 post-approval studies were imposed. In total, 81 initial relative effectiveness assessments (REAs) and 13 reassessments were available, with numbers of drugs (re)assessed varying greatly between jurisdictions. Study results were available for 16 initial REAs (20%) and included in 14 (88%), and available for 10 reassessments (77%) and included in all (100%). Five reassessments had an outcome different from the initial REA, with 4 (2 positive and 2 negative changes) relating directly to the new study results. Reassessments often cited the inability of post-approval studies to resolve the concerns reported in the initial REA.

**Conclusion:** Results from regulator-imposed post-approval studies for conditionally approved drugs were not often used in REAs by HTA organizations, because they were often not yet available at the time of initial assessment and because reassessments were scarce. When available, results from post-approval studies were almost always used within HTA, and they have led to changes in conclusions about drugs’ relative effectiveness. Post-approval studies can be relevant within HTA but the current lack of alignment between regulators and HTA organizations limits their potential.

## Background

**Key Messages**

** Implications for policy makers**

- Post-approval study results were not often included in initial relative effectiveness assessments (REAs) by health technology assessment (HTA) organizations, because initial REAs were usually already finished when post-approval study results became available.
- Post-approval study results were almost always included in REA reassessments, but REA reassessments were scarce.
- Post-approval study results have affected the outcomes of REA reassessments in certain cases either because they resolved major concerns or because they were not able to confirm the prospected relative benefits from the initial REA.
- Regulators and HTA organizations should coordinate post-approval study requirements to improve the relevance of regulator-imposed post-approval studies for HTA.
- HTA organizations should develop processes to systematically evaluate whether and when reassessments are needed and to better align with post-approval regulatory processes.

** Implications for the public**

Innovative drugs can receive a conditional marketing authorization (CMA) based on relatively limited evidence. After conditional approval, the European Medicines Agency (EMA) obligates the manufacturer to perform more studies. Such studies can produce results that may also be relevant for reimbursement authorities. This study shows that such post-approval study results were often not available at time of initial reimbursement decision-making. When they were available they were often used. In reimbursement reassessments (n = 13), if post-approval study results were available (10/13; 77%), they were always included (10/10; 100%). In some (5/10; 50%) of these cases, the reports of reimbursement agencies stated that inclusion of new study results warranted changes to reimbursement recommendations. Patients could benefit more from results of these studies when the EMA and reimbursement agencies align their processes.

To enable timely access to innovative drugs, the European Medicines Agency (EMA) can conditionally approve drugs based on a less comprehensive evidence package when immediate availability of the drug outweighs the risks due to the less comprehensive evidence package.^1,2^ Importantly, the benefit-risk balance still needs to be judged positive, but more uncertainty may be considered acceptable in light of the drug’s potential to address unmet medical needs.

 What constitutes a ‘less comprehensive evidence package’ is to some extent clarified by EMA guidelines and can be related to small sample sizes, surrogate primary endpoints, short follow-up times, and limited safety data, amongst others.^1^ Indeed, research has shown that the evidence package available at approval for drugs with a conditional marketing authorization (CMA) is less comprehensive compared to drugs approved with a standard marketing authorization. A lower percentage of drugs has an evidence package including randomized, controlled and/or blinded studies. Fewer patients are included in the pivotal studies for conditionally approved drugs and fewer studies include clinical primary endpoints.^3-5^ To address these remaining uncertainties and to ensure that more comprehensive evidence is ultimately available, the EMA obligates manufacturers to perform post-approval studies called ‘specific obligations’ (SOBs).

 However, after approval patient access to innovative drugs can remain limited in case of negative reimbursement decisions. To inform decisions regarding a drug’s optimal reimbursement status and level, health technology assessment (HTA) organizations evaluate the benefits of drugs compared to jurisdiction-specific alternative treatments.^6^ These evaluations include relative effectiveness assessments (REAs), together with other HTA considerations. Since the evaluation of efficacy – and, to a lesser extent, safety – by regulators has similarities to REAs performed by HTA organizations, both organizations exhibit similar preferences regarding evidence suitable for their assessments.^7-12^ Nevertheless, the acceptance of less comprehensive evidence for CMA drugs by regulators may not be acceptable for reimbursement decision-making by HTA organizations.^13^ Ideally, regulators and HTA organizations would coordinate their post-approval evidence needs so that results from SOBs can inform HTA reassessments. It is currently unclear to what extent post-approval evidence has informed HTA decision-making.

 Eighty-seven percent of CMA drugs approved between 2006 and 2016 did not receive unrestricted positive reimbursement recommendations.^14^ Negative reimbursement recommendations lead to patient access being delayed, limited or entirely absent, depending on the jurisdiction. For this reason, regulators and HTA organizations have emphasized the relevance of alignment of their processes and evaluations.^15^ Although some HTA organizations have processes in place to conditionally reimburse drugs, these processes are currently not aligned with the EMA conditional approval pathway. Considering that SOBs are in place to ensure comprehensive evidence becoming available, their results could affect reimbursement recommendations and subsequent patient access. However, the execution of SOBs takes time.^16,17^ Thus, results from SOBs may be particularly relevant for HTA reassessments as opposed to initial evaluations. The extent to which results of post-approval studies inform HTA recommendations has never been studied.

 Thus, this study investigated if results from regulator-imposed post-approval studies (ie, SOBs) for conditionally approved drugs were used by HTA organizations within REAs and if so, how these studies have affected the assessments.

## Methods

###  Included Drugs and Jurisdictions

 A retrospective analysis of EMA and HTA reports was performed. All drugs conditionally approved between March 2006 (the start date of the CMA scheme) and December 2018 were included. Included HTA organizations were major European HTA jurisdictions that systematically publish full initial HTA reports and reassessment reports on their websites in a language understood by the investigators, being: England + Wales (National Institute for Health and Care Excellence, NICE), France (Haute Autorité de Santé, HAS), the Netherlands (Zorginstituut Nederland, ZIN) and the European Network for Health Technology Assessment (EUnetHTA). HTA reports were retrieved by searching agencies’ websites for the drug generic and brand name and were included until June 2019, to allow time for HTA decision-making after drug approval. Vaccines were excluded because HTA organizations assess vaccines differently from other drugs.

###  Data Extraction

 To investigate the role of SOBs in REAs, data was extracted for regulatory evaluations and HTA initial assessments and reassessments.

 We recorded general characteristics of drugs including drug name, indication, therapeutic category, orphan status at conditional approval, CMA date (European Commission decision), marketing authorization conversion date (if applicable), and whether the drug had undergone accelerated assessment by the EMA. Drug regulatory data on pivotal observational and interventional studies submitted for approval and to fulfil post-approval SOBs were retrieved from the European public assessment reports. The number of pivotal studies evaluated for approval of the drug and the included primary endpoints within these pivotal studies were recorded. Primary endpoints were categorized as surrogate or clinical efficacy endpoints, or safety endpoints based on the information provided by the European public assessment report and on previous literature describing the type of endpoints in pivotal studies for conditionally approved drugs.^18^

 Considering post-approval studies, all SOBs were extracted from EMA documents following previously published procedures.^17^ The number of SOBs per drug and their original due dates and final submission dates (if applicable) were recorded. The objective of the SOB (addressing, efficacy, safety or other), type of obligation (clinical trial or other) and its status at approval were also recorded. Again, the primary endpoints for those SOBs entailing clinical trials were categorized as surrogate, clinical or safety.

 Data on HTA considerations and conclusions regarding relative effectiveness were retrieved from published HTA reports – including initial assessments as well as reassessments – each matching the initial CMA indication. HTA recommendations that were not substantiated by a consideration of the clinical evidence were excluded (eg, a negative recommendation because no dossier was submitted by the manufacturer). When the CMA concerned multiple indications that were considered separately by HTA organizations, all were included independently. The same approach was applied when HTA organizations split a single indication into recommendations for 2 or more subpopulations. From HTA reports, the dates of the initial assessment and reassessments were recorded, as well as the outcome of the REA and whether the assessments included a discussion of the (lack of) results from completed SOBs.

###  Data Analysis

 First, descriptive statistics were used to describe drug, pivotal study and SOB characteristics.

 Second, based on the dates of included HTA reports, SOB results were categorized as being available for HTA organizations (y/n) in initial REAs as well as in reassessments and it was analyzed whether available SOB results were included by HTA organizations. For initial REAs, the proportions of positive and negative recommendations were compared between those REAs including SOB results and those not including SOB results. To that end, the outcomes of the REAs were categorized into lesser effectiveness, equal effectiveness and higher effectiveness compared to jurisdiction-specific alternative treatments, in line with previous work.^19,20^ When REAs did not include SOBs even though they were already available at the time of HTA decision, it was assessed whether the REA process was already ongoing when SOB results became available. If so, these indications were categorized as having no SOBs available yet.

 Finally, the contributing role of SOB results was assessed by investigating the initial and reassessment REA reports for those drugs that had initial assessments that did not include results from SOBs while the reassessments did. HTA organizations’ major concerns on the clinical evidence were extracted from the reports’ summary statements. From the reassessment reports, statements were extracted about SOB results affecting the assessments and/or assessment outcomes by resolving or not resolving any or all of the major concerns. Major concerns were – in line with previous work – classified into categories related to the trial validity, the patient population, comparative effects, and the relevance of the endpoints and the drug’s effect size on those endpoints.^21-23^ Possible changes to REA outcomes were assessed based on the REA categories used within each jurisdiction.

## Results

###  Characteristics of Included Drugs, Pivotal Studies and Specific Obligations

 Forty drugs have been conditionally approved between January 2006 and December 2018. Three of them were vaccines, and one of them was not assessed by any HTA organization, giving a final cohort of 36 drugs. Table 1 shows characteristics of these drugs and associated pivotal studies and SOBs. The majority of drugs (53%) were approved based on a single pivotal study. In total, 59 pivotal trials supported the drug approvals. The EMA imposed 98 SOBs for the 36 included drugs. For 17 drugs (47%), only 1 SOB was imposed.

**Table 1:** Drug and Trial Characteristics of the 36 Included Drugs

	**No. (%)**
**Drug Characteristics**	
Therapeutic category (based on ATC code)	
Alimentary tract and metabolism	1 (3)
Systemic hormonal preparations	1 (3)
Anti-infectives	6 (17)
Antineoplastic agents	23 (64)
Musculo-skeletal system	2 (6)
Nervous system	2 (6)
Sensory organs	1 (3)
Orphan designation at conditional approval	22 (61)
Converted to standard marketing authorization at 31-12-2018	19 (53)
Number of pivotal trials per drug	
1	19 (53)
2	11 (31)
3	6 (17)
Number of drugs with at least one study with a clinical primary endpoint at conditional approval	1 (3)
Number of SOBs per drug	
1	17 (47)
2	9 (25)
3	2 (6)
4	4 (11)
5	2 (6)
≥6	2 (6)
Number of drugs with SOBs with clinical primary endpoints	9 (25)
**Characteristics of Pivotal Trials**	
Total number of pivotal trials	59
Endpoints included in pivotal trials	
Clinical primary endpoints	1 (2)
Surrogate primary endpoints	56 (95)
Safety endpoints	2 (3)
**Characteristics of SOBs**	
Total number of SOBs	98
SOBs fulfilled at 31-12-2018	77 (79)
SOB is meant to provide insight in	
Efficacy	5 (5)
Efficacy and safety	75 (77)
Safety	8 (8)
Other	10 (10)
Type of SOB	
Clinical trial (final analysis)	66 (67)
Clinical trial (interim analysis)	11 (11)
Other	21 (21)
Status of clinical trials as SOBs at approval (N = 77)	
Already ongoing	50 (65)
New study	27 (35)
Endpoints included in clinical trials as SOBs (N = 77)	
Clinical primary endpoints	13 (17)
Surrogate primary endpoints	57 (74)
Safety primary endpoints	7 (9)

Abbreviations: SOB, specific obligation; ATC, Anatomical Therapeutic Chemical.

###  Inclusion of Health Technology Assessment Reports

**Figure 1:**
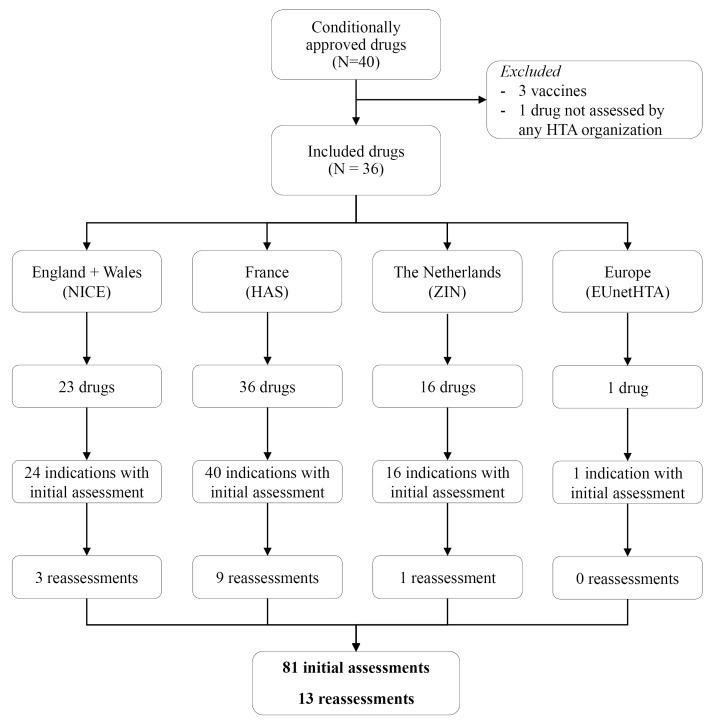
Inclusion Flowchart for the REAs of All Included HTA Organizations. Abbreviations: REAs, relative effectiveness assessments; HTA, health technology assessment; NICE, National Institute for Health and Care Excellence; HAS, Haute Autorité de Santé; ZIN, Zorginstituut Nederland; EUnetHTA, European Network for Health Technology Assessment.

Figure 1 shows the inclusion flowchart of HTA reports for all 36 drugs. In total, 94 HTA recommendations were included, of which 81 were initial assessments and 13 were reassessments. HAS evaluated all drugs, but all other jurisdictions evaluated only a part of the cohort. NICE evaluated 23 drugs, ZIN 16 and EUnetHTA only 1 drug. There was a second report from EUnetHTA (for pazopanib), but the report emphasized that it was not suited for decision-making as it was used to test the EUnetHTA core model. It was therefore excluded from this study. In one occasion NICE split the indication into 2 recommendations. This was the case for 4 drugs for HAS. Reassessments were available for 3 indications (13%) for NICE, 9 (23%) for HAS and for 1 indication (6%) for ZIN. Figure S1 shows the outcomes of the initial REAs of the included HTA organizations (see Supplementary file 1).

###  Availability and Inclusion of Specific Obligations in REAs

**Figure 2:**
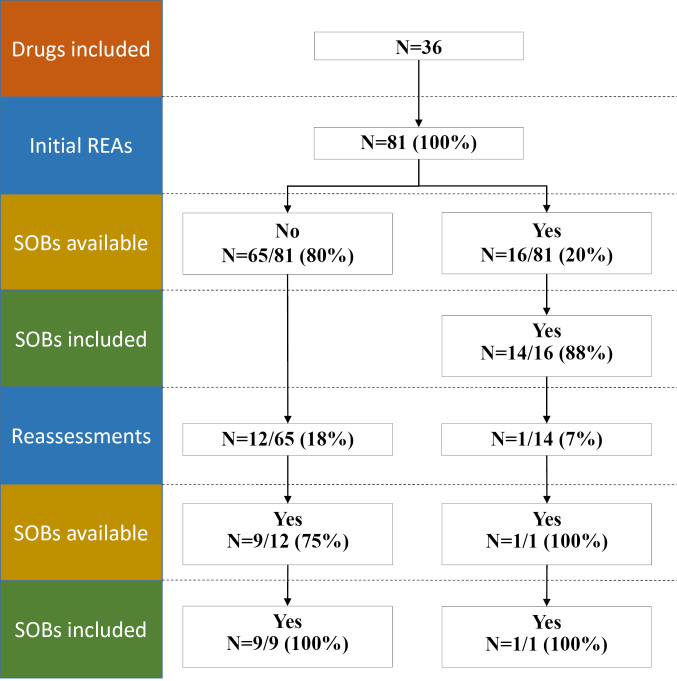
Availability for and Inclusion of SOBs in Initial and Subsequent REAs. Abbreviations: REAs, relative effectiveness assessments; SOBs, specific obligations.

**Figure 3:**
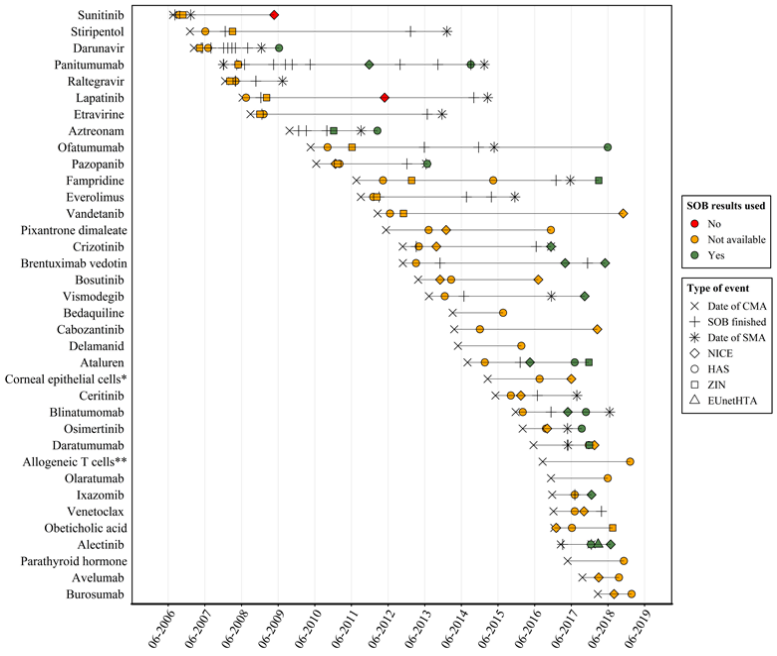
Timeline of Drug Regulatory Decisions and HTAs for Conditionally Approved Drugs. The colors indicate whether results from SOBs were considered during HTA. Abbreviations: HTA, health technology assessment; CMA, conditional marketing authorization; SMA, standard marketing authorization; SOB, specific obligation;NICE, National Institute for Health and Care Excellence; HAS, Haute Autorité de Santé; ZIN, Zorginstituut Nederland; EUnetHTA, European Network for Health Technology Assessment.
 * Ex vivo expanded autologous human corneal epithelial cells containing stem cells (Holoclar®). ** Allogeneic T cells genetically modified with a retroviral vector encoding for a truncated form of the human low affinity nerve growth factor receptor (ΔLNGFR) and the herpes simplex I virus thymidine kinase (HSV-TK Mut2) (Zalmoxis®). HAS performed nine reassessments, NICE three, and ZIN only one.

Figure 2 shows a flowchart of the inclusion of SOBs in initial REAs and in reassessments. SOB results were available for 16 (20%) of 81 initial REAs. Of these 16, 14 (88%) included the available SOB results. SOB results were available for 10 (77%) of all 13 reassessments. All 10 (100%) included those SOB results. Overall, SOBs were included in 24 of 26 cases where they were available (92%). For one of the 10 reassessments that included SOB results the initial assessment already included those results. The availability and inclusion of SOBs in REAs is graphically presented in Figure 3. It shows for all 36 drugs the major events within regulation (conditional and standard marketing authorization, SOB completion dates) and HTA (assessments and reassessments). For HTA events, the figure also indicates whether the EMA SOBs were considered in the assessment (yes/no, if available). Nineteen drugs had their CMA converted to a standard marketing authorization. For 4 of these drugs, an HTA reassessment existed that was not already ongoing at the time of conversion (3 from HAS and 1 from ZIN). All 4 included the available SOB results.

 The median time from CMA to standard marketing authorization was 1095 days (N = 19, IQR = 572-1909) The median time from CMA to initial HTA recommendation was 520 days for NICE (N = 23, IQR = 245-1416), 219 days for HAS (N = 36, IQR = 144-410), 249 days for ZIN (N = 16, IQR = 110-523), and 372 days for EUnetHTA (N = 1). The median time from conversion to a standard marketing authorization to reassessment was 176 days for HAS (N = 3, IQR = 142-1132) and 283 days for ZIN (N = 1).

 Outcomes of the initial REAs seemed similar between the drugs for which SOB results were available and included (N = 14) and those for which they were either available but not included or not available at all (N = 67); 10/14 (71%) versus 43/67 (64%) were positive and 2/14 (14%) versus 11/67 (16%) were negative, see Figure S2.

###  Role of Specific Obligation Results in Relative Effectiveness Assessments

 To assess the role of results from SOBs on REA reassessments, dossiers were analyzed for drugs for which the initial REA did not include results from SOBs while the reassessment did. As shown in Figure 2, of all 13 reassessments, nine met this criterion. Table 2 shows the initial assessment and reassessment outcomes for all nine drugs and the main concerns that impacted the result of the REA.

**Table 2:** Initial and Reassessment REA Outcomes and Main Critique Points of HTA Organizations for the 9 Drugs for Which SOB Results Became Available Between the Initial Assessment and the Reassessment

	**Drug**	**HTA Organization**	**Assessment**	**Relative Benefit**	**Trial Validity**	**Population**	**Comparator**	**Effect Size/Endpoints**
Higher	Osimertinib	HAS	Primary	Absent				
			Reassessment	Minor				
	Pazopanib	HAS	Primary	Insufficient				
			Reassessment	Absent				
Lower	Ataluren	HAS	Primary	Minor				
			Reassessment	Absent				
	Blinatumomab	HAS	Primary	Moderate				
			Reassessment	Minor				
	Ofatumumab	HAS	Primary	Absent				
			Reassessment	Insufficient				
No change	Crizotinib	NICE	Primary	Positive				
			Reassessment	Positive				
	Darunavir	HAS	Primary	Moderate				
			Reassessment	Moderate				
	Fampridine	ZIN	Primary	Negative				
			Reassessment	Negative				
	Panitumumab	HAS	Primary	Absent				
			Reassessment	Absent				

Abbreviations: REAs, relative effectiveness assessments; SOBs: specific obligations; HTA, health technology assessment; NICE, National Institute for Health and Care Excellence; HAS, Haute Autorité de Santé, France; ZIN, Zorginstituut Nederland. HAS = France, NICE = England + Wales, ZIN = The Netherlands. HAS categories that equal a positive REA (higher effect) are minor, moderate and substantial benefit. Absent for HAS means equal effectiveness and insufficient means a negative REA (less effective). The red color means a negative impact of this aspect on the REA and the green color a positive impact. Grey stands for this aspect not being discussed as a main critique point.

 Higher relative benefit at reassessment versus initial assessment was established for 2 drugs: osimertinib (Tagrisso^®^) and pazopanib (Votrient^®^), by HAS. In each case a lack of established comparative effects was the main factor impacting the initial assessment. Therefore, non-inferiority could not yet be established for pazopanib and superiority not for osimertinib. The results from the imposed SOBs established non-inferiority for pazopanib and superiority —although only minor — for osimertinib.

 Lower relative benefit at reassessment versus initial assessment was established for 3 drugs: ataluren (Translarna^®^), blinatumomab (Blincyto^®^) and ofatumumab (Arzerra^®^), by HAS. For ataluren, the initial assessment explicitly stated that even though there were major concerns, the drug was given the benefit of the doubt due to a lack of alternatives. The SOB results did not resolve the concerns of HAS. For blinatumomab, the prospective benefit for a patient population with a medical need was established as moderate awaiting a comparative trial. The SOB resolved this lack, but the effects were judged as less impressive than expected, resulting in a conclusion of minor benefit. For ofatumumab, the SOB did not resolve any of the major concerns, but in the meantime alternative treatments had been approved for the same indication which led to downgrading of the benefit of ofatumumab.

 Equal relative benefit at reassessment versus initial assessment was established for 4 drugs: crizotinib (Xalkori^®^) by NICE, darunavir (Prezista^®^) and panitumumab (Vectibix^®^) by HAS, and fampridine (Fampyra^®^) by ZIN. For 2 (crizotinib and darunavir), the concerns were not regarded as major, resulting in positive REA conclusions in the initial assessments. The SOB results, based on longer follow-up of the pivotal trials at approval, did not change that. Notably, the NICE reassessment of crizotinib considered the longer follow-up of overall survival data, but used this mostly to update the cost-effectiveness model. For the other 2 drugs (panitumumab and fampridine), it was explicitly mentioned that the SOB results did not resolve the major concerns even though for both drugs the SOBs included a newly initiated study.

## Discussion

 This study aimed to investigate if results from regulator-imposed post-approval studies (ie, SOBs) for conditionally approved drugs were used by HTA organizations within REAs and if so, how these studies have affected reassessments.

 Our findings indicate that HTA organizations almost always included results of SOBs for conditionally approved drugs in their assessments, if those results were available at the time of assessment. However, these were only available in a minority of cases, because most initial REAs were performed before any results from SOBs were available. Furthermore, because HTA reassessments were relatively uncommon, most results from SOBs that became available after the initial HTA recommendation were not used within any REA.

 In those cases where SOB results became available between the initial assessment and a reassessment, they had variable effects on HTA recommendations. In 4 cases (44%), SOB results directly led to reassessment conclusions that were different from the initial REA. A lack of established comparative effects was most often the major concern resolved by SOBs. In each case these concerns were resolved through newly initiated studies rather than continuations or extensions of pivotal trials. Depending on how convincing the effect sizes were in the SOB results in relation to what was hypothesized in the initial REA, the relative benefit was either upgraded or downgraded in the reassessment. In the other 5 cases, SOB results did not change the REA. In 2 cases this was because there were no major concerns to be solved by the SOB and in the other 3 cases the SOB results did not adequately resolve the major concerns from the initial REA. For one of those 3 cases, the reassessment REA outcome was nonetheless different from the initial REA, due to factors independent of the assessed drug or the SOB results.

###  Implications

 The lack of initial REAs that included SOB study results was expected given the sequence and timing of regulatory evaluations and HTAs in the drug lifecycle: most initial REAs are already finished by the time any post-approval study results become available. Current initiatives between the 2 stakeholders regarding data sharing and parallel evaluations will likely further shorten the timing between regulatory evaluations and HTA.^15,24^ To ensure incorporation of relevant post-approval study results in HTA decisions, a more systematic approach to reassessments by HTA organizations could therefore be appropriate. Currently, there is a clear misalignment between both stakeholders regarding post-approval processes. Regulators review the CMA annually and aim to ultimately convert the CMA status to a standard marketing authorization, while HTA reassessments of relative effectiveness are scarce and rarely timed after the moment of conversion to standard marketing authorization.

 Our results also indicate that large differences are present in the (re)assessment procedures of the included HTA organizations. HAS aims to evaluate all drugs, while NICE and ZIN have risk-based selection procedures to decide which drugs they will assess. HAS has a procedure for reassessments that dictates reassessments every 5 years, or when new evidence warrants it. However, the reassessments performed by HAS for our cohort of drugs often included only an assessment of the actual benefit (to determine whether the drug should remain on the positive reimbursement list), while no reevaluation of relative effectiveness was performed. Therefore we could not include these reassessments in our analysis. Similarly, NICE can set a date for reassessment during the initial evaluation when this is warranted, or, if no date is set, checks for new evidence every 5 years. Again, for the drugs included in our analysis often NICE screened the evidence and found a reassessment was not necessary. ZIN can reevaluate drugs, and had a reassessment procedure for a selection of (expensive) inpatient drugs from 2006-2014. No systematic reassessment procedure currently exists and reassessments are rare. Reassessments can also be requested by manufacturers, but because many initial REAs are already positive, there may not be many. Indeed, in our study, for most indications for which a reassessment was performed, the initial REA indicated a lack of or little added benefit. There might also be an underreporting of reassessments when REA outcomes remain unchanged. Other factors, for example capacity restraints, may also contribute to the scarcity of reassessments. Further development of targeted reassessment processes – in line with the timing of evidence development and conversion of conditional to standard marketing authorization – for all HTA organizations can facilitate alignment between HTA reassessments and the CMA process of the EMA. Though EUnetHTA assessments for conditionally approved drugs were found to be extremely rare, EUnetHTA has evaluated some CMA drugs approved after the inclusion timeframe of this study (eg, polatuzumab vedotin and crizanlizumab). Besides joint assessments, EUnetHTA may play an important role in the standardization of reassessment processes throughout Europe. A good starting point may be the EUnetHTA report on the criteria to select and prioritize health technologies for additional evidence generation. Full alignment on reassessment processes is nevertheless unlikely for the near future because reassessments may also be triggered by cost aspects or by revisions to national confidential pricing arrangements or treatment guidelines.

 The changes in HTA recommendations as a consequence of the availability of results from SOBs indicate that post-approval evidence can be relevant for HTA organizations. However, our study also indicates that in some cases worries about the quality or relevance of SOB results limited their impact. Lack of study quality or inadequacies in the patient populations, comparators or endpoints have been shown to result in evidence not being helpful for the assessment of relative effectiveness.^22^ Previous studies have also highlighted that data requirements from HTA organizations often go beyond requests made by the EMA.^25^ Early agreement between regulators and HTA organizations regarding appropriate post-approval study requests could lead to study results that are more helpful for HTA organizations.^26^ It has already been shown that regulators and HTA organizations can agree on the most appropriate characteristics for pre-approval studies.^27,28^ Possibly, a similar coordinated approach throughout the entire drug lifecycle could facilitate post-approval evidence generation. However, firm conclusions about the potential impact of post-approval study results are impossible due to the small number of HTA reassessments.

 The adequate and timely completion of post-approval studies can be another area for coordination. Research has shown that SOBs are often delayed, changed, or not finished at all.^16,17^ Coordination between regulators and HTA organizations regarding timing and content of (re)evaluations could provide incentives for timely finishing SOBs. Such coordination requires HTA organizations to be free to vary their timing of reassessments.

 This study focused solely on REAs, but HTA organizations have repeatedly emphasized that the limited evidence associated with conditionally approved drugs does not justify their high prices.^29,30^ Post-approval studies could influence the cost-effectiveness estimate as well as the uncertainty in that estimate by providing more information regarding the drug’s relative effects. Indeed, the availability of more long-term results within the crizotinib reassessment of NICE together with a renegotiation of the drug price led to the overall reimbursement recommendation going from negative to positive, even though the REA had been positive from the beginning. Already, many HTA organizations individually experiment with conditional financing schemes, but the results are mixed and their implementation is uncoordinated across countries.^31^ European coordination between regulators and HTA organizations could result in a joint definition of the necessary evidence to turn a conditional approval and conditional, limited reimbursement into a standard approval and full reimbursement.

###  Limitations

 Precise descriptions of SOBs are often not (publicly) available at the time of marketing authorization, which means that sometimes we could not determine some characteristics of these studies such as the type of endpoint included. Additionally, SOBs are sometimes changed or added in annual renewal procedures of the CMA.^17^ These alterations are not explicitly reported in the public domain which means that we may have missed some SOBs. Our inclusion criteria led to a selection of HTA organizations that do not necessarily represent all HTA organizations in Europe. Most HTA organizations did not systematically publish their full dossiers in a language understood by the investigators. For these reasons, some major jurisdictions were excluded from our study (eg, Germany, Italy and Spain) and our results cannot readily be extrapolated to these or other jurisdictions. Last, for the timeline in Figure 3, we identified decision dates or, when these were not reported, dossier publication dates, which are arguably a bit later than the actual decision dates.

## Conclusion

 Results from post-approval studies for conditionally approved drugs are not often used in REAs by HTA organizations, mostly because they are not yet available at the time of assessment. However, when they are available they are almost always used, and they have led to changes in the conclusions about drugs’ relative effectiveness. Coordination of the post-approval evidence needs between regulators and HTA organizations, increased oversight over the finishing of post-approval studies, and a more systematic approach to reassessments by HTA organizations may facilitate appropriate patient access to conditionally approved drugs.

## Ethical issues

 Ethical approval was not required for this study because no patient data has been used. All data included in this study is publicly available.

## Competing interests

 HGML reports that he is a member of the Lygature Leadership Team. The other authors declare no support from any organization for the submitted work and no financial relationships with any organizations that might have an interest in the submitted work in the previous three years. All authors report no other relationships or activities that could appear to have influenced the submitted work.

## Authors’ contributions

 RAV, LTB, SvO, WGG, and AKMT devised and planned the study. RAV, LTB and SvO conducted the data extraction and analysis. RAV, LTB, JH, HGML, OHK, MEE, WGG, and AKMT contributed to the interpretation of the results. RAV and LTB drafted the first manuscript. All authors reviewed and revised the manuscript in subsequent iterations. All authors approved the final version of the manuscript.

## Disclaimer

 The views expressed in this article are the personal views of the authors and may not be understood or quoted as being made on behalf of or reflecting the position of the agencies or organizations with which the authors are affiliated.

## Authors’ affiliations

^1^Division of Pharmacoepidemiology and Clinical Pharmacology, Utrecht Institute for Pharmaceutical Sciences, Utrecht University, Utrecht, The Netherlands. ^2^The National Health Care Institute, Diemen, The Netherlands. ^3^Dutch Medicines Evaluation Board, Utrecht, The Netherlands. ^4^Department of Innovation Studies, Copernicus Institute of Sustainable Development, Utrecht University, Utrecht, The Netherlands. ^5^Faculty of Health Sciences, University of Southern Denmark, Odense, Denmark.

## Supplementary files


Supplementary file 1 contains Figures S1-S2.
