# Supplementary material for: The Role of Regulator-Imposed Post-Approval Studies in Health Technology Assessments for Conditionally Approved Drugs
Source: Int J Health Policy Manag. 2020 Oct 27;11(5):642–50. doi: 10.34172/ijhpm.2020.198 (PMC9309934; doi:10.34172/ijhpm.2020.198)
Supplement: Supplementary file 1 — contains Figures S1-S2. [file ijhpm-11-642-s001.pdf]

Supplementary file 1

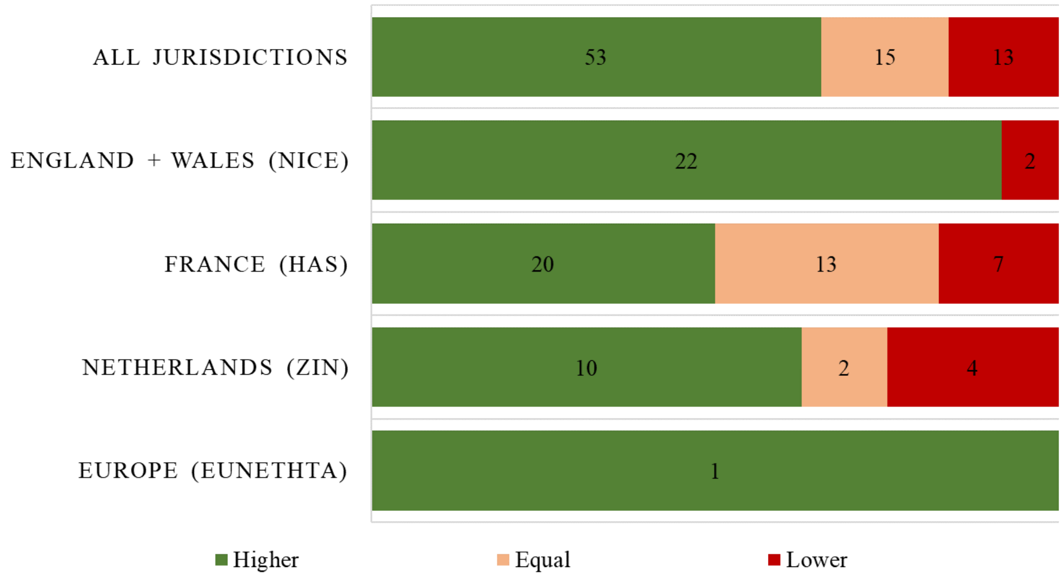

Figure S1. Primary relative effectiveness assessment outcomes (N=81) for the included drugs (N=36), excluding not assessed drugs. NICE split an indication into two recommendations in one case and HAS in 4 cases.

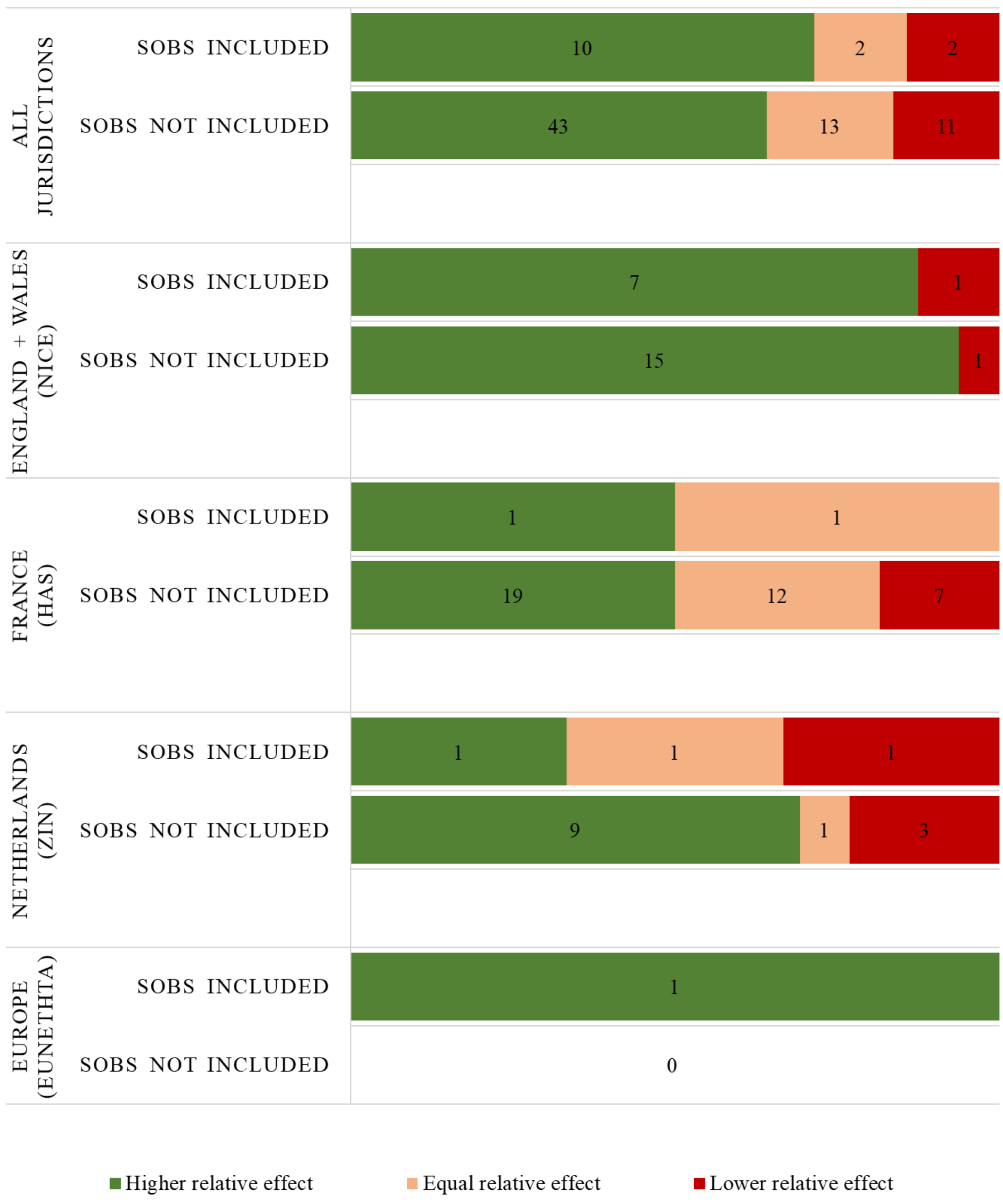

Figure S2. Outcomes of the initial relative effectiveness assessments with specific obligation results that were available and included versus relative effectiveness assessments that did not include specific obligations because they were either not available or not included. SOB: specific obligation.
